# Hematemesis in Children: A Potential Sign of Parental Psychiatric Disorder

**DOI:** 10.7759/cureus.61985

**Published:** 2024-06-09

**Authors:** Madiha Benhachem, Hassnae Tkak, Aziza Elouali, Abdeladim Babakhouya, Maria Rkain

**Affiliations:** 1 Department of Pediatrics, Faculty of Medicine and Pharmacy, Mohammed VI University Hospital, Mohammed First University, Oujda, MAR

**Keywords:** mortality, bleeding, abuse, psychiatric disorder, munchausen’s syndrome

## Abstract

Munchausen’s syndrome by proxy (MSBP) is a rare form of abuse characterized by the fabrication or induction of symptoms of illness in a child by a close relative, typically a parent, leading to multiple consultations and varying degrees of invasive medical interventions. Various clinical presentations are described in the literature, ranging from organic manifestations to psychiatric expressions. This syndrome remains a challenging diagnosis to make and requires increased awareness among healthcare professionals. Prompt recognition is key to preventing potential long-term comorbidities and even fatalities. Here, we are reporting two cases of MSBP manifested by bleeding, with the perpetrator being the mother.

## Introduction

Munchausen’s syndrome by proxy (MSBP) is a factitious disorder simulated and/or induced by a parent. The child is repeatedly and persistently presented for medical care, leading to multiple and repeated medical and surgical investigations. Traditionally, symptoms regress when the child is separated from the responsible parent, but this sign is not always confirmed. In MSBP, the most common physical symptoms include bleeding, predominantly hematuria, convulsions, and loss of consciousness. Clinicians must remain vigilant in the face of atypical or recurrent medical presentations without clear explanation as MSBP can remain undetected and have severe repercussions on the child's welfare [1].

## Case presentation

Case No. 1

**Figure 1:**
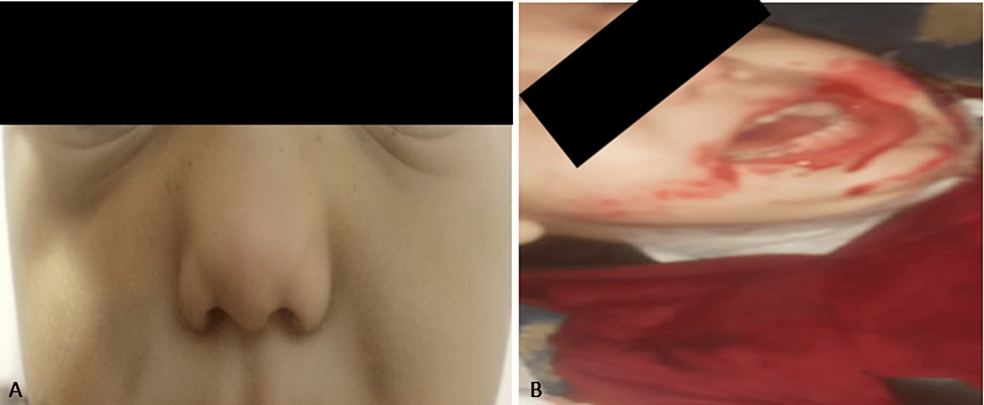
A: Bilateral perinasal ecchymoses and B: Presence of blood around the mouth

A 19-month-old male infant, presented by his mother to the emergency department for recurrent hemorrhagic syndrome since the age of seven months, characterized by hematemesis, with a history of multiple consultations and hospitalizations for the same reason, requiring blood transfusion and treatment with proton pump inhibitors. Clinical examination revealed an overall healthy but pale infant, with perinasal ecchymoses (Figure 1).

**Figure 2:**
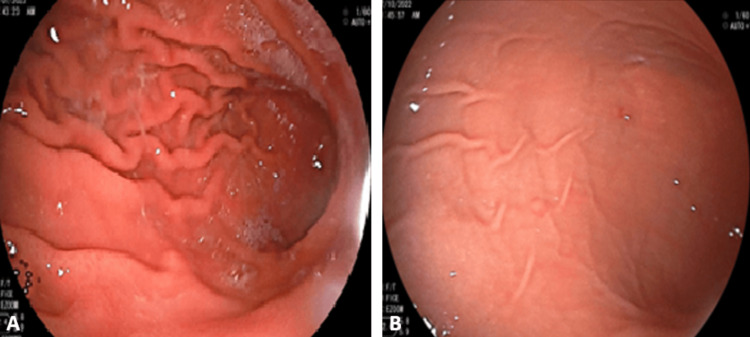
Upper gastrointestinal endoscopy performed for Case No. 1 (A) and for Case No. 2 (B) reveals no signs of bleeding

Several additional examinations were conducted, some of which were repeated multiple times, revealing iron deficiency anemia. Hemostasis tests showed no abnormalities (Table 1). Upper gastrointestinal endoscopy and nasofibroscopy did not reveal any signs of bleeding (Figure 2).

**Table 1:** Biological findings of our patients (Case Nos. 1 and 2)

Laboratory parameter	Test results case No. 1	Test results case No. 2	Reference range
Haemoglobin (g/dL)	7.1	6.1	10.5-13.5
Mean corpuscular volume (fL)	59.3	62	80-98
Mean corpuscular hemoglobin concentration (%)	17.5	16.6	27-32
White blood cell (/µL)	9600	10,500	5200-11,000
Lymphocyte (/µL)	4280	3800	2300-5400
Neutrophil (/µL)	3460	5680	1500-7000
Platelets (/µL)	497,000	596,000	150,000-400,000
Reticulocyte (/µL)	8900	12000	20,000-80,000
Prothrombin time (%)	95	100	70-100
Activated partial thromboplastin time: patient/control	1	1	0.8-1.2
Fibrinogen (g/L)	2.6	2.9	2-4
Ferritinemia (ng/mL)	4.7	6	15-150

During hospitalization, the child experienced several bleeding episodes reported only by the mother, including bilateral epistaxis, hematemesis (Figure 1), hematuria, and blood in the diapers. However, tests for blood in urine and stool were negative. Subsequently, the patient had a generalized tonic-clonic seizure in an afebrile context, and laboratory tests revealed hyperkalemia at 7.1 mEq/L. Further investigation into this hyperkalemia revealed that the infant had ingested potassium gluconate given by the mother, which was found in her possession. Given this clinical polymorphism and the normality of paraclinical investigations, MSBP was suspected, and psychiatric consultation was requested for the mother.

Case No. 2

**Figure 3:**
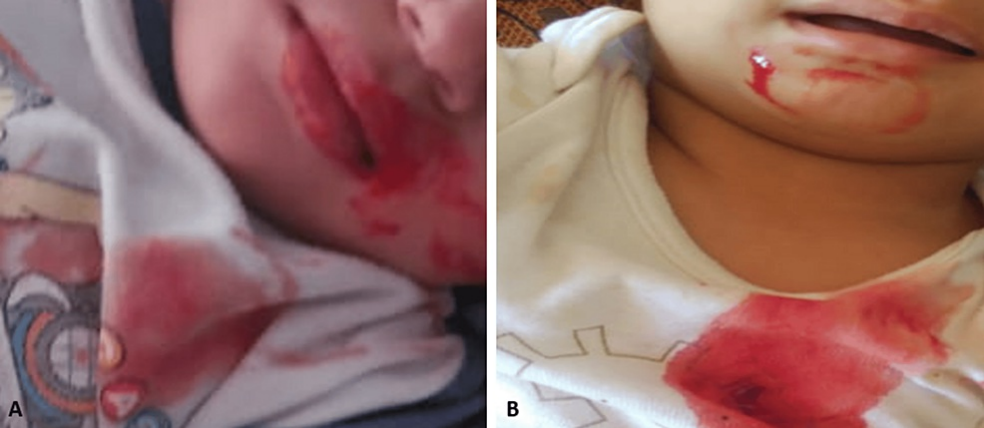
Images of bleeding (A and B)

A 14-month-old male infant was admitted for upper gastrointestinal bleeding characterized by hematemesis for the past week. Clinically, he did not present any specific signs apart from cutaneous-mucous pallor. Biochemically, he had iron deficiency anemia; hemostasis tests were normal (Table 1), and upper gastrointestinal endoscopy and nasofibroscopy revealed no abnormalities. During the period of hospitalization, the infant experienced a second episode of hematemesis (Figure 3), prompting a second endoscopic exploration, which yielded normal findings (Figure 2). One month later, the infant was readmitted for a hemorrhagic syndrome consisting of isolated otorrhagia without any history of trauma. The otorhinolaryngological examination and biological tests were normal.

Given the normality of the clinical examination, as well as the exhaustive paraclinical assessment conducted, MSBP is ultimately suspected, especially considering the mother's particular psychiatric profile.

## Discussion

MSBP is a rare and complex disorder known since 1977, with the first case reported by Meadow [2]. It is described as a factitious disorder that can be simulated or induced by a parent, usually the mother, leading to frequent consultations with repeated and persistent medical examinations and treatment prescriptions. This syndrome is characterized by the parent's denial of the cause of the symptoms, which regress after the child is separated from the responsible parent [1].

MSBP accounts for 0.04% of child abuse cases [3], with infants being the primary victims due to their inability to articulate the abuse suffered, while children over six years old represent only 25% [4].

The presence and support of the abusive parent, especially during hospitalizations, make diagnosing Munchausen syndrome very challenging. Several conditions can serve as warning signs to suspect this syndrome, including unexplained persistence of symptoms requiring repeated hospitalizations and investigations, the ineffectiveness of administered treatments, the disappearance of symptoms in the absence of the abusive parent, a parent less concerned than healthcare professionals, a parent refusing to separate from their child, and a history of unexplained deaths within the family [5-7].

The clinical presentation is highly variable and nonspecific, characterized by the repetition of the same symptoms. The main symptoms include consciousness and balance disorders, seizures, apnea, bleeding (hematemesis, rectal bleeding, hematuria, epistaxis, and hemoptysis), diarrhea, vomiting, intestinal obstructions, hypoglycemia, fever, dehydration, and skin rashes. MSBP can also manifest as factitious psychiatric disorders, including alleged behavioral disorders, mood disorders, schizophrenia, and post-traumatic stress disorder [1,8-10].

The two cases described in our department presented with induced organic disorders by the mother, with a history of bleeding. Yates et al. published the first focused review on perpetrators [11], analyzing 250 articles; according to this review, the mother was the perpetrator in 95.58% of cases, with a health-related profession reported in 45.55% of the cases. Borderline personality disorder was the most commonly described personality disorder, and factitious disorder imposed on another was reported in 30.9% of cases.

MSBP carries a mortality risk of up to 10%, stemming from the abuse inflicted by the parent or excessive medical interventions [8]. In addition to this tragic outcome, serious sequelae such as academic delay, social integration difficulties, post-traumatic stress disorder, and even the development of factitious disorders are also observed [8,12]. It is imperative to emphasize the lethal consequences and morbidities associated with delayed diagnosis of this syndrome and to underscore the paramount duty of physicians to safeguard the child from the earliest signs of suspicion.

## Conclusions

MSBP remains a rare and poorly understood etiology of hematemesis in children. The detection of MSBP relies on the discrepancy between the child's recurrent symptoms and normal examination results, prompting suspicions of manipulation. The complex diagnosis requires collaboration among healthcare professionals, social services, and sometimes legal authorities to protect the child, implement safety measures, provide family support, and ensure necessary medical care.
